# Discovery of a Novel Microtubule Targeting Agent as an Adjuvant for Cancer Immunotherapy

**DOI:** 10.1155/2018/8091283

**Published:** 2018-10-10

**Authors:** Fumi Sato-Kaneko, Xiaodong Wang, Shiyin Yao, Tadashi Hosoya, Fitzgerald S. Lao, Karen Messer, Minya Pu, Nikunj M. Shukla, Howard B. Cottam, Michael Chan, Dennis A. Carson, Maripat Corr, Tomoko Hayashi

**Affiliations:** ^1^Moores Cancer Center, University of California San Diego, La Jolla 92093, USA; ^2^School of Pharmaceutical Sciences, Shenzhen University Health Science Center, Nanhai Ave 3688, Shenzhen, Guangdong 518060, China; ^3^Division of Biostatistics, University of California San Diego, La Jolla 92093, USA; ^4^Department of Medicine, University of California San Diego, La Jolla 92093, USA

## Abstract

For an activating immunotherapy such as adjuvants, a compound that can prolong immune stimulation may enhance efficacy. We leveraged data from two prior high throughput screens with NF-*κ*B and interferon reporter cell lines to identify 4*H*-chromene-3-carbonitriles as a class of compounds that prolonged activation in both screens. We repurchased 23 of the most promising candidates. Out of these compounds we found** #1** to be the most effective agent in stimulating the release of cytokines and chemokines from immune cells, including murine primary bone marrow derived dendritic cells. Mechanistically,** #1** inhibited tubulin polymerization, and its effect on immune cell activation was abolished in cells mutated in the beta-tubulin gene (*TUBB*) encoding the site where colchicine binds. Treatment with** #1** resulted in mitochondrial depolarization followed by mitogen-activated protein kinase activation. Because tubulin polymerization modulating agents have been used for chemotherapy to treat malignancy and** #1** activated cytokine responses, we hypothesized that** #1** could be effective for cancer immunotherapy. Intratumoral injection of** #1** delayed tumor growth in a murine syngeneic model of head and neck cancer. When combined with PD-1 blockade, tumor growth slowed in the injected tumor nodule and there was an abscopal effect in an uninjected nodule on the contralateral flank, suggesting central antitumor immune activation. Thus, we identified a new class of tubulin depolymerizing agent that acts as both an innate and an adaptive immune activating agent and that limits solid tumor growth when used concurrently with a checkpoint inhibitor.

## 1. Introduction

Activation of dendritic cells (DCs) is one of the critical first steps in developing an antigen specific adaptive immune response. The NF-*κ*B pathway is upregulated after antigen presenting cell (APC) activation and inflammatory cytokines are produced [1, 2]. As a safeguard to prevent excessive inflammation which could be deleterious to the host, negative feedback molecules are subsequently induced, which then attenuate these processes [3, 4]. Therapies aimed at activating the immune system (vaccinations and tumor immunotherapy) require stimulation of APCs but could benefit from overcoming negative feedback loops to sustain signaling of NF-*κ*B and other pathways through the time required for the APCs to traffic to draining lymph nodes.

Hence, we aimed to identify small molecular weight compounds that enhanced and prolonged innate immune stimulation by using two high throughput screens (HTS) with THP-1 reporter cell lines for NF-*κ*B [5] and interferon activation [6]. The THP-1 NF-*κ*B-*bla* cell lines and the THP-1 ISRE-*bla* cell lines are equipped with a beta-lactamase reporter gene under the control of an NF-*κ*B response or an ISRE response element, respectively. We stimulated these reporter cell lines with lipopolysaccharide (LPS) or human type I interferon (IFN) as primary stimulators and concurrently with 166,000 individual compounds to screen for extended reporter activity after 12 h or 16 h incubation [5, 6]. Analysis of the results revealed that compounds with a 4*H*-chromene-3-carbonitrile scaffold exhibited an immune phenotype of preventing deactivation or prolonging the activation of both NF-*κ*B by LPS at 12 h and ISRE at 16 h [5]. An agent with the ability to prolong the key signaling pathways critical to the function of APCs, NF-*κ*B or type I IFN would be advantageous as an immune stimulating adjuvant.

While most adjuvants target innate immune receptors, such as Toll-like receptors (TLR) and intracellular nucleotide receptors, recent studies have also indicated other potential targets, including microtubules. Microtubules interact with many intracellular proteins and regulate protein trafficking of multiple signaling pathways [7–13]. Clinically, microtubules have been a direct target for anticancer drugs because of their roles in mitosis and cellular dynamics [14–16]. Recent reports indicate that specific microtubule depolymerization agents induce immunogenic cell death of cancer cells and improve outcomes of cancer immunotherapy [17, 18]. Some of these agents can directly stimulate innate immune responses and activate murine DCs by upregulating maturation-markers and by induction of proinflammatory cytokines [7, 19].

Despite the potential of microtubules as a promising target, few chemically distinct compounds have been brought forward as immune adjuvants. In this study, we characterize a small molecule with a 4*H*-chromene-3-carbonitrile scaffold, which was confirmed to induce and sustain NF-*κ*B activation and to induce cytokine production by primary murine APCs and a human monocytic cell line (THP-1). This compound triggered mitochondrial stress via inhibition of microtubule polymerization and induced mitogen-activated protein kinases (MAPK) activation. We further demonstrate that intratumoral administration of the lead compound both reduced tumor growth in a murine syngeneic head and neck cancer model and enhanced an immune response with concurrent use of anti-PD-1 antibodies.

## 2. Materials and Methods

### 2.1. Mice

Six- to eight-week-old C57BL/6 mice were purchased from the Jackson Laboratories (Bar Harbor, MA). All animal experiments received prior approval by the UCSD Institutional Animal Care and Use Committee (IACUC).

### 2.2. Cells and Reagents

THP-1 NF-*κ*B-*bla* cell lines were purchased from Thermo Fisher Scientific (Waltham, MA). THP-1 ISRE-*bla* cell lines were developed by Thermo Fisher Scientific [6]. THP-1 cells were purchased from ATCC (American Type Culture Collection, Manassas, VA). Cells were cultured in RPMI medium (Thermo Fisher Scientific, Waltham, MA) supplemented with 10% FBS (Omega Scientific Inc., Tarzana, CA), 100 U/mL penicillin, 100 *μ*g/mL streptomycin (Thermo Fisher Scientific), and 55 *μ*M *β*-mercaptoethanol (Sigma-Aldrich, St. Louis, MO). Mouse primary bone marrow derived dendritic cells (BMDCs) were generated from bone marrow cells harvested from the femurs of C57BL/6 mice as previously described [20, 21].

The human T cell lymphoblast cell line, CEM, was obtained from ATCC. The CEM-178 cell line has a point mutation in the* TUBB* gene. The mutation caused a lysine to arginine substitution at amino acid 350 in the *β*-tubulin molecule making it resistant to microtubule-depolymerizing agents [22]. B16-OVA cells are a melanoma line transfected to express ovalbumin (OVA) [23]. Wild type mouse tonsil epithelial cells (MTECs) that are human papillomavirus- (HPV-) negative [24, 25] were a gift from Dr. John Lee (Sanford Research, Sioux Falls, South Dakota, USA) and were cultured in E-media, which consisted of 68% DMEM (Thermo Fisher Scientific), 23% Ham F12 (Thermo Fisher Scientific), 10% FBS and supplemented with 500 *μ*g/L hydrocortisone (Sigma-Aldrich), 8.4 *μ*g/L cholera toxin (Sigma-Aldrich), 5 *μ*g/L transferrin (Sigma-Aldrich), 5 *μ*g/L insulin (Sigma-Aldrich), 1.36 *μ*g/L tri-iodo-thyronine (Sigma-Aldrich), and 5 *μ*g/L epidermal growth factor (EGF) (Thermo Fisher Scientific). HPV-negative MTECs were tested for mouse pathogen contamination (IMPACT II mouse test, IDEXX BioResearch, Columbia MO) prior to being introduced into mice. LPS-EB Ultrapure (InvivoGen, San Diego, CA), JNK inhibitor S600125 (Selleckchem, Houston, TX), and DMSO (Sigma-Aldrich) were used for* in vitro* experiments. Rat anti-mouse PD-1 monoclonal antibody (mAb) (clone, RMP1-14, BioXcell, West Lebanon, NH) and rat IgG2a isotype control (clone 2A3, BioXcell) were used for* in vivo* experiments. Colchicine was purchased from Sigma Aldrich. Other compounds were purchased from ChemBridge Corporation and ChemDiv and were verified for purity (>98%) by LC-MS. Endotoxin levels of all compounds were determined by Endosafe (Charles River Laboratory, Wilmington, MA) and were less than 10 EU/*μ*mol.

### 2.3. Assay Using THP-1-Blue™ NF-*κ*B Cells

The THP-1-Blue™ NF-*κ*B cell line has a stable integration of an NF-*κ*B-inducible secreted embryonic alkaline phosphatase (SEAP) reporter construct and was purchased from InvivoGen (#thp-nfkb). The levels of NF-*κ*B-induced SEAP in the cell culture supernatant were measured using QUANTI-Blue, a SEAP detection reagent (#rep-qbl, InvivoGen) according to the manufacturer's protocol.

### 2.4. Immunoblot

THP-1 cells were rested overnight and then treated with 5 *μ*M** #1** in the presence or absence of 4 ng/mL LPS for indicated time periods. Cells were lysed with PhosphoSafe Extraction Reagent (EMD Millipore, Billerica, MA) supplemented with protease inhibitor cocktail (Roche, Manheim, Germany) and 0.1% SDS (Thermo Fisher Scientific). Protein concentrations were measured using Pierce BCA protein assay kit (Thermo Fisher Scientific). Ten *μ*g of reduced and denatured protein per sample was then separated by gel electrophoresis and transferred onto PVDF membranes. Membranes were blocked with 5% nonfat milk in 0.1% Tween-20 Tris-buffered saline (TBST) and subsequent washes were done in TBST. Primary antibody and secondary antibody were diluted in 5% bovine serum albumin- (BSA-) TBST and 5% nonfat milk-TBST. Anti-phospho NF-*κ*B p65 (#3033), anti-NF-*κ*B p65 (#8242), anti-I*κ*B*α* (#4814), anti-phospho JNK (#9251), anti-JNK (#9252) anti-*β*-actin (#3700), anti-rabbit (#7074), and anti-mouse IgG HRP-linked antibodies (#7067) were all purchased from Cell Signaling Technology (Danvers, MA).

### 2.5. Immunofluorescent Staining

THP-1 cells were treated with 5 *μ*M** #1** in the presence or absence of 4 ng/mL LPS for 2 and 8 h. After treatment, cells were fixed for 20 min at room temperature with 3.7% formaldehyde and then incubated in 0.1% Triton X-100-PBS for 20 min and incubated in 3% BSA-PBS for 30 min at room temperature. After rinsing with PBS, cells were stained with anti-NF-*κ*B p65 rabbit monoclonal antibody overnight at 4°C (1:800, #8482, Cell Signaling Technology). After rinsing with PBS, cells were incubated with goat anti-rabbit IgG Alexa Fluor 488 for 1 h at room temperature (1:600, #111-545-144, Jackson ImmunoResearch, West Grove, PA). After washing in PBS, cells were mounted in anti-fade reagent with DAPI (4′,6-diamidino-2-phenylindole) (#P36931, Thermo Fisher Scientific) and images were captured using an Axio Imager (Zeiss, Germany).

### 2.6. Gene Expression Analyses

QuantiGene Plex Assay kits for* IL8*,* IL1B*,* CCL2,* and* IL23A* were provided by Affymetrix (Santa Clara, CA). 10^6^ cell/mL of THP-1 cells were treated with 5 *μ*M** #1** with 10 ng/mL LPS. After 4 and 16 h treatment, the culture supernatants were removed, and cells were lysed in lysis buffer. Assays were performed according to the manufacturer's protocols. For quantitative RT-PCR, RNA was extracted from THP-1 cells using RNA extract kit (Zymo Research, Irvine, CA) and was reverse-transcribed using iScript (Bio Rad, Hercules, CA). TaqMan Gene Expression assays (Thermo Fisher Scientific) were performed using CFX-Connect Real-Time System using primers for* HPRT* (Hs02800695_m1),* IL1B* (Hs01555410_m1),* IL8* (Hs00174103_m1)* CCL2* (Hs00234140_m1), and* IL23A* (Hs00372324_m1) as previously described [26].

### 2.7. ELISA and Cell Viability Assay

Mouse BMDCs, THP-1 cells, CEM cells, or* TUBB* mutant CEM-178 cells [22] were treated with compound (0.1 or 5 *μ*M) or vehicle in the presence or absence of LPS overnight. A portion of the supernatant was transferred and secreted levels of KC, IL-6, IL-12, and IL-1*β* were assessed by ELISA according to the manufacturer's protocol (R&D systems). 0.5 mg/mL 3-[4,5-dimethylthiazol-2-yl]-2,5-dipheyl tetrazolium bromide (MTT, Thermo Fisher Scientific) solution was added to each well and incubated 6 h and formazan crystals were then lysed with lysis buffer (15% SDS and 0.12% 12 N HCl). The absorbance was measured at 570 nm using 650 nm as a reference with a plate reader (Tecan, Switzerland).

### 2.8. Tubulin Polymerization Assay

Tubulin polymerization was tested by a tubulin polymerization assay kit (#BK011P, Cytoskeleton Inc., Denver, CO) in the presence of vehicle (DMSO), 3 *μ*M compound, or colchicine according to the manufacturer's protocol.

### 2.9. RNA Sequencing

THP-1 cells were incubated for 5 h with vehicle control or 5 *μ*M** #1** alone and then RNA was isolated. High throughput RNAseq was performed by the sequencing core at La Jolla Institute for Allergy and Immunology (San Diego, CA). Briefly each experimental group had 3 replicates. RNAseq was used to obtain gene expression values. Reads were mapped using TopHat with hg19 annotation; reads mapped to tRNA/rRNA, adapters, and intergenic regions were filtered; HTseq was used for read count quantification. Genes were filtered if more than 2/3 of the samples had counts <10. About 13,500 genes were included in final analyses. Raw counts were then upper-quantile normalized and used as expression values in the following analysis. Linear models for microarray (Limma, using R-limma package) were used to compare groups regarding log2 expression values. The Benjamini-Hochberg procedure was applied to control the false discovery rate (FDR). A gene was considered significantly changed if FDR <0.05. If log_2_ fold change in expression for a test compound versus control was greater than 0, it was said to be upregulated; otherwise, it was downregulated. In addition, log_2_ fold changes between two groups of interest (e.g.,** #1** versus vehicle) for all the genes included in the analysis set were extracted and preranked for gene set enrichment analysis (GSEA, using GSEA v3.0 from the Broad Institute). Enrichment in KEGG pathways (c2.cp.kegg.v6 from Broad Institute) was examined. For each gene set, the number of expressed genes in the analysis dataset was calculated along with enrichment score, the p value and q value for testing enrichment significance, and the number of core enrichment genes.

### 2.10. Cell Cycle Analysis

2.5 × 10^5^ cells/mL THP-1 cells were cultured with 5 *μ*M** #1** overnight. Cells were fixed in 70 % ethanol for 5 min at room temperature. After washing with PBS, cells were stained with 10 *μ*g/mL of DAPI for 15 min at room temperature and analyzed by flow cytometry (Miltenyi Biotec, Germany). Data was analyzed with FlowJo software (FlowJo, Ashland, OR, USA).

### 2.11. Mitochondria Stress Assay

THP-1 cells, WT CEM cells, and CEM-178 cells were treated with 0.1 or 5 *μ*M** #1**, colchicine, and vinblastine for 72 h. MitoTracker Red FM (Thermo Fisher Scientific) was used according to the manufacturer's protocol to examine depolarized mitochondria by flow cytometry.

### 2.12. Control of Established Tumor Growth and Conditional Survival

2 x 10^6^ HPV-negative MTECs in 50 *μ*L were implanted in both flanks of C57BL/6 male mice. When the diameter of the tumors reached 2-4 mm (approximately day 7), treatment was initiated. 20-200 nmol** #1** or vehicle (10% DMSO) was injected intratumorally in a total volume of 50 *μ*L and anti-PD-1mAb (250 *μ*g/injection) or rat IgG2a isotype control was intraperitoneally injected in a volume of 100 *μ*L. Tumors were measured on two perpendicular axes using a caliper. Estimates of tumor volume (mm^3^) were calculated using ([width]^2^ x [length]/2). Mice were euthanized if the tumor diameter reached 15 mm or the tumor ulcerated in accordance with UCSD IACUC guidelines.

### 2.13. Statistical Analysis

Data are presented as means with standard error of mean (SEM) or standard deviation (SD). For analysis of* in vitro* data, one-way ANOVA with Tukey's test was used to compare multiple groups and a two-tailed Mann-Whitney* U* test was used to compare two groups. Two-way ANOVA with a Bonferroni corrected* post hoc* test was used for the* in vivo* data. The log rank (Mantel-Cox) test was used to test for a significant difference between Kaplan-Meier survival curves. Prism 5 software (GraphPad Software, San Diego, CA) was used; all comparisons are two-sided at 5% significance level.

## 3. Results and Discussion

### 3.1. Structure-Activity Relationship Studies in 4*H*-Chromene-3-Carbonitriles

**Figure 1:**
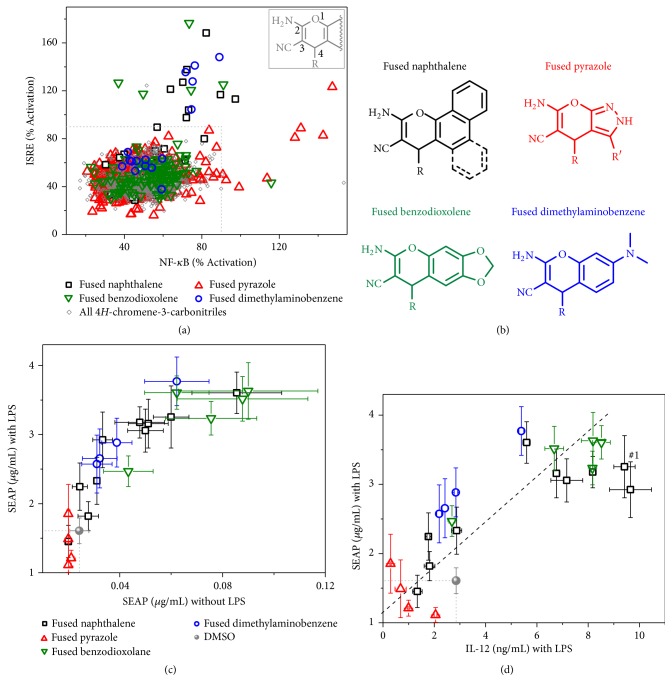
**Structure-activity relationship studies in 4*H*-chromene-3-carbonitriles.** (a) HTS identification of the 4*H*-chromene-3-carbonitriles (insert) as a hit scaffold. We reanalyzed the data from two prior HTS for prolongation of NF-*κ*B activation 12 h after LPS versus ISRE activation 16 h after IFN*α* administration with a chemical library containing 1,778 compounds within this scaffold. The %activation values of each compound relative to the LPS and IFN*α* controls in the original HTS, respectively, are shown. (b) Structures for fused naphthalene, fused pyrazole, fused benzodioxolane, and fused dimethylaminobenzene scaffolds. (c) SEAP production by the THP-1-Blue NF-*κ*B reporter cell line stimulated with 5 *μ*M of each compound in the absence versus the presence of 10 ng/mL LPS. Each symbol indicates the means ± SEM for triplicates of individual compounds. (d) IL-12 secretion by BMDCs stimulated with compounds in the presence of 0.5 ng/mL LPS significantly correlated with SEAP production measured by THP-1-Blue NF-*κ*B reporter cells measured in triplicate. The dotted line indicates the regression line (Spearman r=0.81, P<0.0001, n=23). Each symbol indicates the means ± SEM for triplicates of individual compounds.

We had previously identified 4*H*-chromene-3-carbonitriles as a class of compounds that prolonged NF-*κ*B activation by LPS [5] in a high throughput screen (HTS) with 166,304 compounds. In a separate HTS we noted that this scaffold was also active in prolongation of an ISRE signal induced by IFN*α* [6]. By reanalyzing the two studies we identified 1,778 compounds with the 4*H*-chromene substructure that were screened in both HTS. We plotted the “% activation” values relative to their respective controls for these compounds from the NF-*κ*B versus the ISRE HTS (Figure 1(a)). We then examined the structural features that are necessary for these potencies in two different immune activating assays. The 4*H*-chromene-3-carbonitriles have two sites of variability (Figure 1(a) inset): first, the site fused to the chromene ring, as shown by wiggly lines and, second, the R group at position 4. Substructural classification by the scaffold fused to the chromene ring yielded 13 major groups (Supplementary Table 1).

Most of the potent compounds belonged to four major groups: fused naphthalene, fused pyrazole, fused benzodioxolane, and fused dimethylaminobenzene, with each group having more than 2 compounds with “% activation” values greater than 90% full activation in either of the HTS screens (Figure 1(a)). The structures for these scaffolds are shown in Figure 1(b), with variable sites marked by R and R'. While the 4*H*-chromene-3-carbitrile scaffolds have been associated with pan-assay interference (PAINS) compounds due to presence of *α*-*β* unsaturated nitriles, it is important to note that the activity here was not related to this functional group as the vast majority of compounds in this series were inactive and only a specific subset of compounds was potent in both screens.

Within each group identified above, the compounds that had a 3-substitution (*meta*) on the phenyl ring and at the 4-position of the chromene were more active compared to the 2-position (*ortho*), while 4-monosubstituted (*para*) phenyls were inactive. Also, within the fused naphthalene group, compounds that were derived from 1-naphthol (solid bonds in black Figure 1(b)) were more active than compounds derived from 2-naphthol (dotted bonds in black Figure 1(b)), which were completely inactive, clearly indicating the structural requirements for activity. Thus, both the angle of the fused group and substitution at the 4-position of the chromene play key roles for activity in prolonging NF-*κ*B activation.

To further evaluate these four chemical groups, we purchased 23 compounds from commercial vendors and confirmed their purity by LC-MS. Among the fused naphthalene derivatives, 10 compounds were purchased including active hits (**#1**-** #8**) and inactive compounds such as 4-isopropyl substituted phenyl (**#9**) and a 2-naphthol derived compound** #10**. In the fused pyrazolo series, derivatives** #11** -** #14** were purchased. Similarly, in the fused dimethylaminobenzene scaffold,** #15** -** #18,** and in the fused benzodioxolane series,** #19** -** #23,** were purchased. The structures for these compounds are shown in Supplementary Figure 1.

### 3.2. 4*H*-Chromene-3-Carbonitriles Activate NF-*κ*B Signaling in the Presence and Absence of TLR4 Stimulation

Immune activities of these 23 compounds were first evaluated using the THP-1-Blue™ NF-*κ*B reporter cell line, which allows quantification of NF-*κ*B activation by SEAP levels in the culture supernatant. Compounds were tested in both the absence and presence of LPS (10 ng/mL). Most of the compounds except the fused pyrazole analogs showed innate stimulatory activity as a single agent as well as enhancement of NF-*κ*B activity with LPS co-stimulation (Figure 1(c) and Supplementary Table 2). Two inactive analogs in the fused naphthalene series were consistent with very low to no activity in this assay. Most of the fused benzodioxolane analogs were highly active, while some of the fused dimethylaminobenzene analogs were only weakly active.

Next, we assayed these compounds in the presence of LPS for NF-*κ*B dependent IL-12 production in primary murine BMDCs, as this is a key cytokine for an APC priming environment (Figure 1(d) and Supplementary Table 2). IL-12 release from BMDCs stimulated by these compounds in the presence of LPS was significantly correlated with NF-*κ*B activation (Figure 1(d), Spearman r=0.81, P<0.0001). Most of the compounds in the fused benzodioxolane series were active except the bis-substituted** #23**. In the fused naphthalene series, besides the inactive analogs, the 2-substituted compound** #2** and naphthyl substituted** #6** did not enhance induction of IL-12, suggesting that 3-substitution (*meta*) on the C4-phenyl of the chromene plays a key role for potency.

### 3.3. Lead Compound Impacts Mitochondrial Function and Cytokine Gene Expression

**Figure 2:**
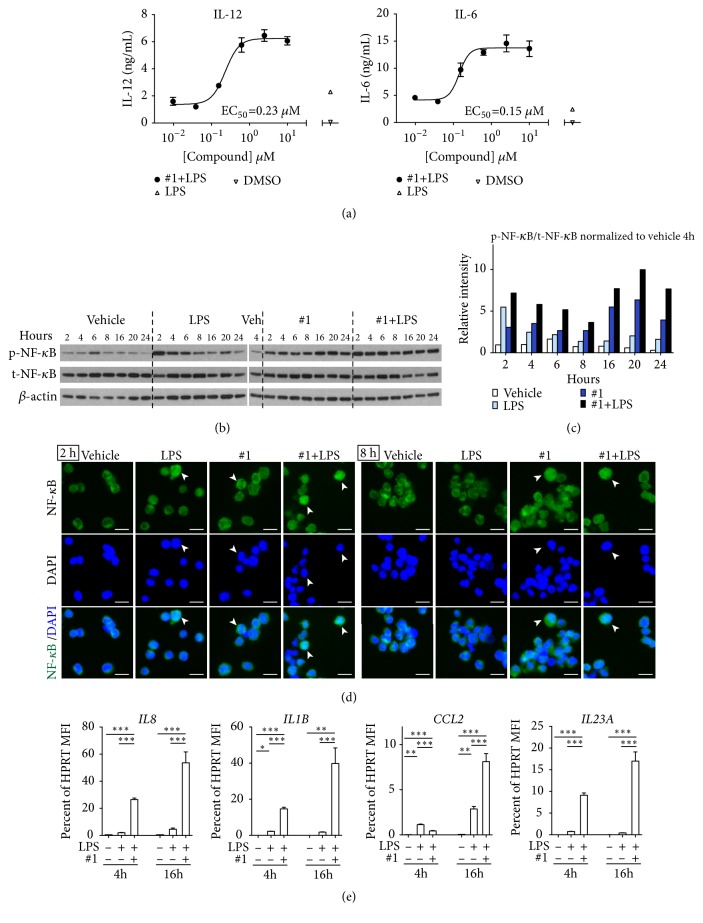
**Lead compound #1, a fused naphthalene, enhances LPS induced cytokine production and sustains NF-** **κ** **B signaling.** (a) EC_50_ for IL-12 and IL-6 production. BMDCs were treated for 18 h with graded concentrations of** #1** (from 10 *μ*M) in the presence of 0.5 ng/mL LPS. 0.5 % DMSO was used as a negative control. IL-12 and IL-6 secretion levels were measured by ELISA. EC_50_ for IL-12 and IL-6 were 0.23 *μ*M and 0.15 *μ*M, respectively. (b) Immunoblot analysis for NF-*κ*B signaling pathway in THP-1 cells. THP-1 cells were treated with compound** #1** (5 *μ*M) in the presence or absence of 4 ng/mL LPS at indicated time periods; p-NF-*κ*B and t-NF-*κ*B indicate phospho-NF-*κ*B and total-NF-*κ*B, respectively. (c) Relative intensity of bands was calculated using ImageJ software. Band intensities were normalized to vehicle 4 h. (d) Nuclear translocation of NF-*κ*B by** #1**. After 2 h and 8 h treatment with 5 *μ*M** #1**, THP-1 cells were fixed and stained for NF-*κ*B (green) and nuclear DNA (blue). The overlap in the merged images appears pale green. Arrowheads indicate nuclear translocation. Bars indicate 10 *μ*m. (e) NF-*κ*B targeted gene expression analysis. THP-1 cells were treated with 5 *μ*M** #1** plus 4 ng/mL LPS for 4 h and 16 h. Expressions of* IL8*,* IL1B*,* CCL2,* and* IL23A* were examined by QuantiGene plex assay and the expression was normalized to* HPRT* mean fluorescence intensity (MFI) (=100). *∗*P<0.05, *∗∗*P<0.01, and *∗∗∗*P<0.0001 by one-way ANOVA with Tukey's* post hoc* test. Data are presented as mean ± SD of triplicate and are representative of two independent experiments showing similar results.

To examine potential intracellular mechanisms that are targeted by the 4*H*-chromene-3-carbonitriles we selected a candidate for a lead compound from the fused napthalene series that potently increased IL-12 and SEAP production in the presence of LPS, compound** #1** (Figure 1(d)). This compound was the most potent at stimulating innate immune cells (BMDC) to produce IL-12 (EC_50_=0.23 *μ*M) and IL-6 (EC_50_=0.15 *μ*M) in the presence of LPS (Figure 2(a) and Supplementary Table 3). We confirmed the effect of compound** #1** on phosphorylation and nuclear translocation of NF-*κ*B in THP-1 cells (Figures 2(b), 2(c), and 2(d)). The phosphorylation of the p65 subunit of NF-*κ*B induced by compound** #1** was sustained for at least 24 h (Figures 2(b) and 2(c)). In THP-1 cells, compound** #1** stimulated NF-*κ*B nuclear translocation at 2 h and 8 h with and without LPS (Figure 2(d)). Gene expression analysis by QuantiGene plex assay showed that NF-*κ*B downstream cytokine genes (*IL8*,* IL1B*,* CCL2,* and* IL23A*) were significantly upregulated in THP-1 cells treated with** #1** in the presence of LPS after 16 h treatment (Figure 2(e)). These results were confirmed by quantitative RT-PCR (Supplementary Figure 2).

After confirming the effects of the lead compound we then explored its impact on the mRNA transcription signature in THP-1 cells by RNAseq analysis. The genes involved in innate immune signaling (“cytokine-cytokine receptor interaction”, “complement and coagulation cascade” and “NOD-like receptor”), NF-*κ*B signaling (“MAPK signaling”), and interferon signaling (“JAK-STAT”) pathways in the KEGG analysis were upregulated by compound** #1** treatment compared to vehicle (Supplementary Figure 3 and Supplementary Table 3). These pathways were all consistent with the original HTS discoveries that compound** #1** is active in regulating NF-*κ*B signaling with TLR4 stimulation and ISRE signaling with type I interferon (Figure 1(a)). We also noted that genes involved in “oxidative phosphorylation” and” DNA replication” pathways were significantly downregulated following** #1** treatment.

### 3.4. 4*H*-Chromene-3-Carbonitriles Inhibit Tubulin Polymerization

The downmodulated KEGG pathways indicated dysregulation of cell division and mitochondrial function. The antiproliferative effects of** #1** were confirmed in THP-1 cells; however, cell cycle analysis indicated an accumulation at the G2/M phase rather than an S phase arrest, suggesting that this compound might target the mitotic spindle (Supplementary Figure 4A and 4B). We searched the literature for prior reports of similar molecules that affected cellular replication. One compound, LY290181, with a 4*H*-chromene-3-carbonitrile scaffold [2-amino-4-(3-pyridyl)-4*H*-naphtho(1,2-*b*) pyran-3-carbonitrile] was described as inhibiting vascular smooth muscle cell proliferation after balloon angioplasty for coronary artery disease [27]. LY290181 was reported to inhibit cell proliferation by binding to tubulin with high affinity and inhibiting microtubule polymerization [28–30]

**Figure 3:**
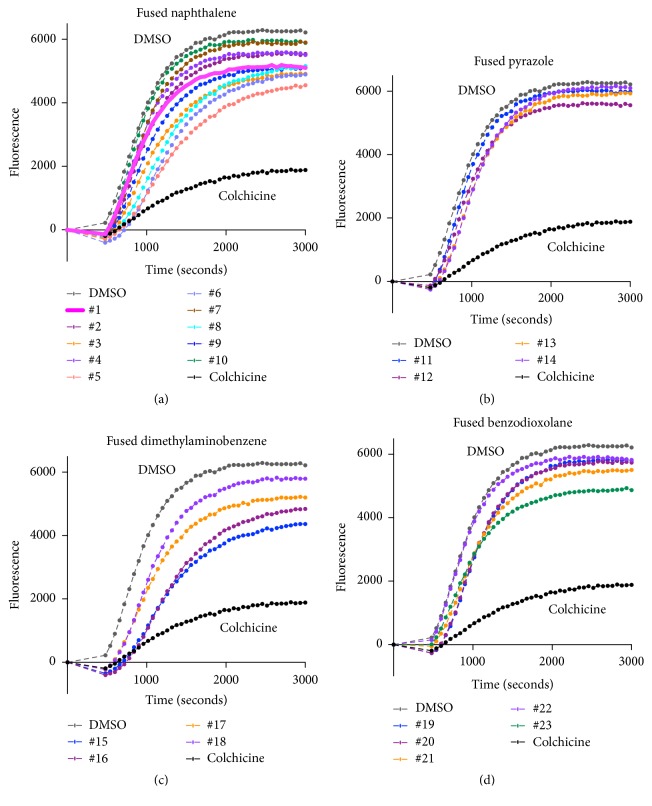
**Inhibition of tubulin polymerization evaluated by cell-free tubulin polymerization assay.** Inhibition of tubulin polymerization by individual compounds (3 *μ*M) or colchicine (3 *μ*M) was measured by a tubulin polymerization assay kit (Cytoskeleton Inc.) according to the manufacturer's protocol. Tubulin polymerization curves are shown following incubation with (a) fused naphthalene, (b) fused pyrazole, (c) fused dimethylaminobenzene, or (d) fused benzodioxolane.

Based on this report and our functional RNAseq data, we examined all 23 4*H*-chromene-3-carbonitrile compounds for their ability to inhibit microtubule polymerization using a cell free fluorescence-based assay (Figures 3(a)–3(d)). Some of the compounds belonging to fused naphthalene and fused dimethylaminobenzene groups showed modest inhibition compared to the colchicine control, while compounds in the fused benzodioxolane and fused pyrazole groups showed minimal if any inhibition. These data suggested that compounds in two classes of derivatives attenuated microtubule polymerization.

### 3.5. Inhibition of Tubulin polymerization Leads to Mitochondrial Stress and Immune Activation

**Figure 4:**
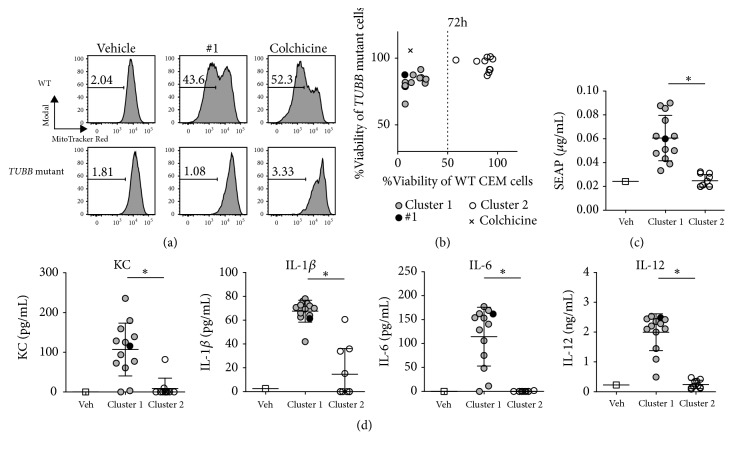
**Tubulin polymerization inhibiting compounds induce mitochondrial stress and show cytotoxicity in WT CEM cells.** (a) Analysis of mitochondrial depolarization. WT CEM cells and* TUBB* mutant CEM-178 cells were treated with 0.1 *μ*M** #1** or colchicine for 72 h and stained with MitoTracker Red, which stains mitochondria in a membrane potential dependent manner. Cells were analyzed by flow cytometry and representative histograms are shown. (b) Cell numbers of WT CEM cells and* TUBB* mutant CEM-178 cells were measured by MTT assay. Cells were cultured with 0.1 *μ*M compounds or 0.01 *μ*M colchicine for 72 h. Each dot indicates an individual compound. Compounds were categorized into two clusters, cluster 1 (< 50% viability; n=13; closed circles) and cluster 2 (> 50% viability; n=10; open circles).** #1** is the closed circle in black. Individual compound IDs are shown in Supplemental Figure 5. (b) NF-*κ*B activation shown in Figure 1(c) was reanalyzed according to the viability clusters. (c) ELISA for KC, IL-1*β*, IL-6, and IL-12 in BMDCs. BMDCs were cultured with 5 *μ*M compounds overnight. *∗*P<0.0001 by Mann-Whitney* U* test for comparison of cluster 1 versus cluster 2. Data presented are representative of two independent experiments showing similar results.

Intracellular assembly of microtubules is crucial for signal transduction and mitosis of cells [12, 13, 31]. In addition, modulation of intracellular microtubule assembly is a well-known cause of mitochondrial stress [32–34]. Hence, we examined mitochondrial membrane potential following addition of compound** #1** to THP-1 cells using MitoTracker Red, a dye that differentially stains mitochondria depending on the membrane potential. Compound** #1** treatment increased mitochondrial depolarization similar to the that of the positive control, colchicine (Supplemental Figure 4C). Subsequently, we assessed the effects of** #1** on mitochondria using a human T cell lymphoblast cell line (CEM) and a* TUBB *mutant CEM cell line (CEM-178), which is mutated in the beta-tubulin gene (*TUBB*) where the colchicine binding site is encoded [22]. The mutant cells were refractory to changes in mitochondrial depolarization induced by** #1** treatment (Figure 4(a)). These data indicated that compound** #1** induced mitochondrial stress associated with inhibition of microtubule polymerization.

To examine the relationship between tubulin polymerization inhibition and immune activation, we treated WT CEM and* TUBB *mutant CEM-178 cells with each compound and assessed cell proliferation after 72 h. We categorized the compounds into two clusters according to reduced cell proliferation seen in WT CEM but not in the* TUBB *mutant CEM (CEM-178): 13 compounds showed less than 50% cell growth in WT CEMs (cluster 1, filled circles) and 10 compounds showed more than 50% (cluster 2, open circles; Figure 4(b) and Supplementary Figure 5). The compounds in cluster 1 induced significantly higher levels of NF-*κ*B activation (Figure 4(c)) and higher levels of IL-1*β*, KC, IL-6 and IL-12 in the culture supernatants of BMDCs compared to compounds in cluster 2 (P< 0.01, Figure 4(d)). These data suggested that compounds in cluster 1 may stimulate DCs through cellular stress.

**Figure 5:**
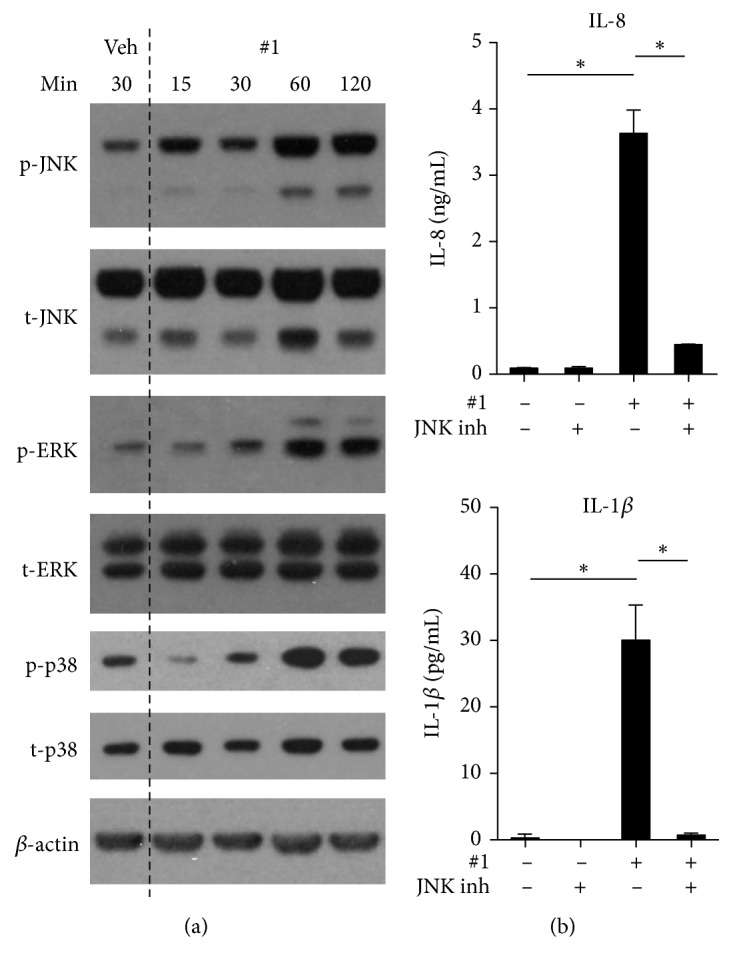
**Compound #1 induces immune activation through MAPK activation**. (a) Immunoblot for activation of MAPK signaling pathways. THP-1 cells were cultured for indicated time periods with 5 *μ*M** #1. **Phospho-JNK (p-JNK), total JNK (t-JNK), phospho-ERK (p-ERK), total ERK (t-ERK), phospho-p38 (p-p38), total p38 (t-p38), and b-actin are shown. (b) Effect of JNK inhibitor on cytokine release in THP-1 cells. Cells were stimulated for 18 h with 5 *μ*M** #1** and 20 *μ*M JNK inhibitor, SP600125. IL-8 and IL-1*β* secretions from THP-1 cells were measured by ELISA. *∗*P<0.0001 by one-way ANOVA with Tukey's* post hoc* test). Data presented are representative of two independent experiments showing similar results.

Mitochondrial stress induced by various environmental stimuli can activate mitogen-activated protein kinases (MAPK) including extracellular signal-regulated kinases (ERK1/2), p38, or c-Jun NH2-terminal kinase (JNK) [35, 36]. Because MAPK signaling participates in the regulation of NF-*κ*B transcriptional activity [37–40], we evaluated phosphorylation of MAPKs in THP-1 cells following** #1** treatment. ERK1/2, p38, and JNK, were phosphorylated in response to treatment with** #1** (Figure 5(a)). Further, to validate the involvement of MAPK in the immunostimulatory effects of** #1**, we evaluated the effect of a JNK inhibitor, S600125, on** #1** stimulated release of IL-8 and IL-1*β*. The levels of IL-8 and IL-1*β* stimulated by compound** #1** were markedly reduced in the presence of S600125 (Figure 5(b)) indicating that MAPK activation and phosphorylation is involved in the innate immune stimulatory effects of this compound. Collectively, the results suggest that** #1** inhibits tubulin, induces mitochondria stress, and leads to MAPK phosphorylation. The combined activation of MAPK and NF-*κ*B can subsequently lead to the cytokine production by APCs that we detected in the BMDC cultures.

### 3.6. Control of Established Tumor Growth and Conditional Survival Using #**1** as Immunotherapy

There are conflicting reports on the effect of microtubular depolymerizing agents on the immune system. Because intracellular microtubules are required for the translocation of intracellular signaling molecules, high concentrations of microtubule depolymerizing agents, such as colchicine, downregulate innate immune responses [15, 41, 42]. In other studies, microtubule depolymerizing drugs used as chemotherapeutic agents, such as vinblastine, have potentiated immune responses [15, 43]. Recently, specific microtubule-depolymerizing agents such as colchicine have gained attention because they have promoted innate and adaptive immune responses during cancer immunotherapy [18, 19, 44]. Therefore, compound** #1** might be useful both to slow the growth of a solid tumor and to stimulate an antitumor immune response. Prior to evaluating the antitumor efficacy, the direct toxicity of** #1** for tumor cells was evaluated* in vitro* at three dosing schedules. MTECs, an epithelial cell line derived as a model of oropharyngeal malignancy [24], were more sensitive to** #1** than B16-OVA cells (a murine melanoma line engineered to express OVA) [23]. In both cell lines the toxicity was increased in a dose and time-dependent manner (Supplemental Figure 6A).

**Figure 6:**
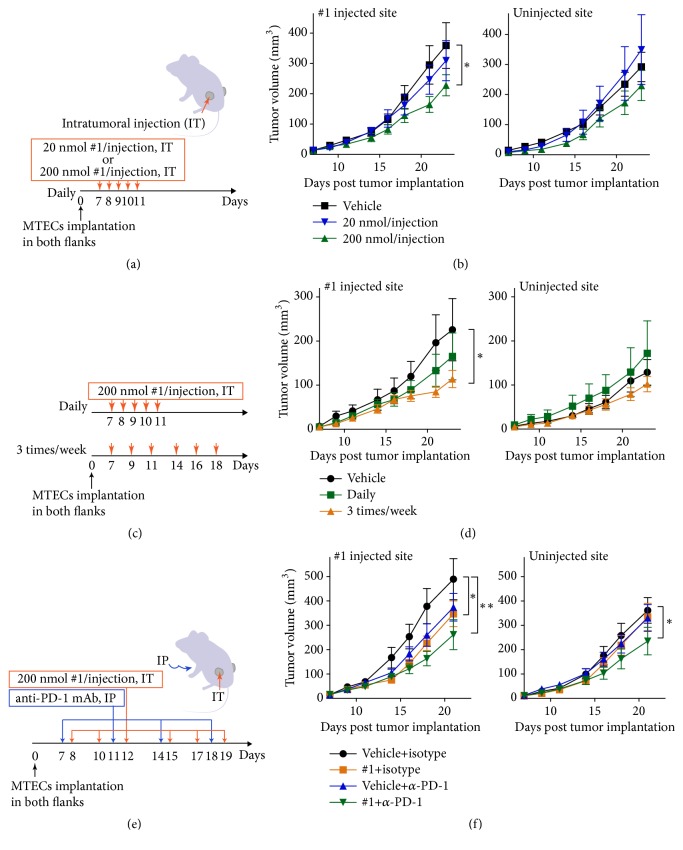
**Combination treatment with #1 and anti-PD-1 mAb suppressed tumor progression at both injected and distal sites.** (a-d) HPV-negative MTECs (2 × 10^6^) were subcutaneously implanted in both flanks of C57BL/6 mice (n=5-7/group). The optimal doses (20 or 200 nmol/injection) and schedules (daily or 3 times/week) of intratumor treatment of compound** #1** were evaluated. Experimental protocols ((a) and (c)) and growth curves of injected and uninjected tumor ((b) and (d)) are shown. ((e) and (f)) Combination treatment with** #1** and anti-PD-1 mAb (n=10-11/group). Tumor bearing mice received intratumoral injection of compound** #1** (200 nmol/injection, 3 times/week) and intraperitoneal administration of anti-PD-1 mAb (250 *μ*g/injection) (e). Average calculated tumor volumes are shown ± SEM (f). *∗*P<0.05, *∗∗*P < 0.001 by two-way ANOVA with Bonferroni* post hoc* test.

To test the antitumor effect of** #1**, we selected the murine head and neck cancer MTECs to implant into mice, as MTECs were shown to be more sensitive to compound** #1** than the B16-OVA cells (Supplementary Figure 6A). To avoid systemic toxicity and to use the tumor as a potential antigenic reservoir for activated APCs, compound** #1** was injected directly into the established tumor nodules. Two doses were used with daily injections of engrafted tumor nodules. The dose of 200 nmol/ injection significantly suppressed growth of the injected nodule compared to 20 nmol/injection (P<0.05, Figures 6(a) and 6(b)). We then determined an optimal treatment schedule using 200 nmol/ injection and different dosing intervals (Figures 6(c) and 6(d)). The mice were treated with** #1** daily for 5 injections or 3 times a week for two weeks (6 injections) and tumor growth at the injected site was monitored. The schedule of 200 nmol injections given three times a week was significantly more effective compared to vehicle treatment in slowing growth of the injected tumor nodule (P<0.05, Figure 6(d)).

Since** #1** activates innate immune cells, we hypothesized that combination therapy with an anti-PD-1 mAb would increase treatment efficacy by releasing T cells from immunosuppression by tumor cells [45]. Compound** #1** was administered for 2 weeks (total 6 injections) (Figure 6(e)) and anti-PD-1 mAb was administered on day -1 followed by injecting twice a week, per an established protocol [45]. At the injected sites, compound** #1** as a single agent significantly suppressed tumor growth on day 18 (P<0.05, Figure 6(f)) and its effect was potentiated by anti-PD-1 therapy (P<0.001, Figure 6(f) and Supplemental Figure 6B). The suppression of tumor growth was also observed at the uninjected site (an abscopal effect), suggesting that the combination treatment induced tumor specific adaptive immune responses. Mice that received combination treatment survived longer than vehicle treated mice (P<0.05, Supplementary Figure 6C). These data suggested that compound** #1** might activate immune cells in the tumor microenvironment and simultaneously induce tumor cell death thereby releasing new tumor antigens and danger signals (Supplementary Figure 7).

## 4. Conclusion

In this study we have demonstrated that compounds of the 4*H*-chromene-3-carbonitrile scaffold containing a fused naphthalene, benzodioxolane, or dimethylaminobenzene group fused to the chromene ring are capable of activating innate immune cells through sustained NF-*κ*B activation to produce inflammatory cytokines. Among active compounds, fused naphthalene, and fused dimethylaminobenzene, substituted compounds showed modest inhibition of microtubule polymerization. The compounds that were the most cytostatic to cells were also the most immunostimulatory. A lead fused naphthalene compound** #1** induced mitochondrial stress, MAPK and JNK activation. Its ability to produce cytokines was blocked by JNK inhibition. Intratumoral administration of** #1** potentiated the anti-tumor activity of anti-PD-1 antibody at an uninjected tumor site indicative of an abscopal effect.

## Data Availability

The RNA-seq data that support the findings of this study was deposited in the ArrayExpress database at EMBL-EBI under accession number E-MTAB-7267.

## Supplementary Materials

Supplementary Materials
** Supplementary Figure** **1**.** Structures of select purchased 4****H****-chromene-3-carbonitriles**.** Supplementary Figure ****2**. Quantitative RT-PCR of gene expression of* IL8*,* IL1B*,* CCL2,* and* IL23A* in THP-1 cells. THP-1 cells were treated with 5 *μ*M** #1** in the presence and absence of LPS for 4 and 16 h. Expressions of* IL8*,* IL1B*,* CCL2,* and* IL23A* were measured by quantitative RT-PCR and the levels were normalized to* HPRT*. *∗*P<0.05, *∗∗*P<0.01, and *∗∗∗*P<0.0001 by one-way ANOVA with Tukey's* post hoc* test.** Supplementary Figure ****3**. RNA-seq data was analyzed by enrichment method using KEGG pathway database. THP-1 cells were incubated with compound** #1** for 5 h and RNA was isolated. RNA-seq was performed by La Jolla Institute Next Generation Sequencing Facility. The most significantly upregulated (upper panels) and downregulated pathways (lower panels) are shown (FDR q<0.05).** Supplementary Figure ****4**. Compound** #1** induces cell cycle arrest at the G2/M phase and mitochondrial depolarization in THP-1 cells. (A) Representative histograms of cell cycle analysis. 5×10^4^ THP-1 cells cultured with 5 *μ*M** #1** or colchicine overnight. After fixation with 70% ethanol, cells were washed with PBS and stained with 10 *μ*g/ml of DAPI for 15 min at room temperature and flow cytometry analysis was performed. (B) Distribution (%) of cells in G1, G2/M and S phases. Data presented are representative of two independent experiments showing similar results. (C) THP-1 cells were treated with 0.1 or 5 *μ*M** #1** or colchicine for 72 h and stained with MitoTracker Red, which stains mitochondria in a membrane potential dependent manner. Cells were analyzed by flow cytometry and representative histograms are shown.** Supplementary Figure ****5**. Detailed compound distribution of Figure 4(b). The individual compound IDs are labeled next to their respective symbols from the plot in Figure 4(b).** Supplemental Figure ****6**. Antitumor effects of compound** #1**. (A) Cytotoxicity of** #1** increased in a time-dependent manner. 10^5^ cells/mL HPV negative MTECs or B16-OVA cells were cultured for 24 h (black), 48 h (green), and 72 h (blue) with serially diluted compound** #1** from 15 *μ*M. At the indicated times cell viability was tested by MTT assay as described in the methods. Comparisons among 24 h, 48 h, and 72 h at each concentration were examined by one-way ANOVA with Tukey's* post hoc* test (*∗*P<0.0001, NS: not significant). (B) Tumor growth curves from individual mice in Figure 6(f) are shown. (C) Kaplan-Meier survival curves of each treatment group are shown. Mice that received combination treatment survived longer compared to vehicle treated mice [*∗*P<0.05 by Log-rank (Mantel-Cox) test].** Supplemental Figure ****7**. Model of functional activity for compound** #1**. Compound** #1** inhibits intracellular beta-tubulin polymerization, which is associated with mitochondrial depolarization and cell cycle arrest. Mitochondrial distress initiates a cascade of MAPK and NF-*κ*B activation which results in cytokine/chemokine release. Intratumor injection of compound** #1** activates APCs in the tumor microenvironment and concurrently induces the release of tumor-antigens, which provides the stimulus of tumor-specific cellular responses.
