# Supplementary material for: Discovery of a Novel Microtubule Targeting Agent as an Adjuvant for Cancer Immunotherapy
Source: Biomed Res Int. 2018 Oct 10;2018:8091283. doi: 10.1155/2018/8091283 (PMC6199861; doi:10.1155/2018/8091283)
Supplement: Supplementary Materials — Supplementary Figure 1. Structures of select purchased 4H-chromene-3-carbonitriles. Supplementary Figure 2. Quantitative RT-PCR of gene expression of IL8, IL1B, CCL2, and IL23A in THP-1 cells. THP-1 cells were treated with 5 μM #1 in the presence and absence of LPS for 4 and 16 h. Expressions of IL8, IL1B, CCL2, and IL23A were measured by quantitative RT-PCR and the levels were normalized to HPRT. ∗P<0.05, ∗∗P<0.01, and ∗∗∗P<0.0001 by one-way ANOVA with Tukey's post hoc test. Supplementary Figure 3. RNA-seq data was analyzed by enrichment method using KEGG pathway database. THP-1 cells were incubated with compound #1 for 5 h and RNA was isolated. RNA-seq was performed by La Jolla Institute Next Generation Sequencing Facility. The most significantly upregulated (upper panels) and downregulated pathways (lower panels) are shown (FDR q<0.05). Supplementary Figure 4. Compound #1 induces cell cycle arrest at the G2/M phase and mitochondrial depolarization in THP-1 cells. (A) Representative histograms of cell cycle analysis. 5×10^4^ THP-1 cells cultured with 5 μM #1 or colchicine overnight. After fixation with 70% ethanol, cells were washed with PBS and stained with 10 μg/ml of DAPI for 15 min at room temperature and flow cytometry analysis was performed. (B) Distribution (%) of cells in G1, G2/M and S phases. Data presented are representative of two independent experiments showing similar results. (C) THP-1 cells were treated with 0.1 or 5 μM #1 or colchicine for 72 h and stained with MitoTracker Red, which stains mitochondria in a membrane potential dependent manner. Cells were analyzed by flow cytometry and representative histograms are shown. Supplementary Figure 5. Detailed compound distribution of Figure 4(b). The individual compound IDs are labeled next to their respective symbols from the plot in Figure 4(b). Supplemental Figure 6. Antitumor effects of compound #1. (A) Cytotoxicity of #1 increased in a time-dependent manner. 105 cells/mL HPV negative MTECs or B16-OV [file 8091283.f1.pdf]

Supplementary Figure 1

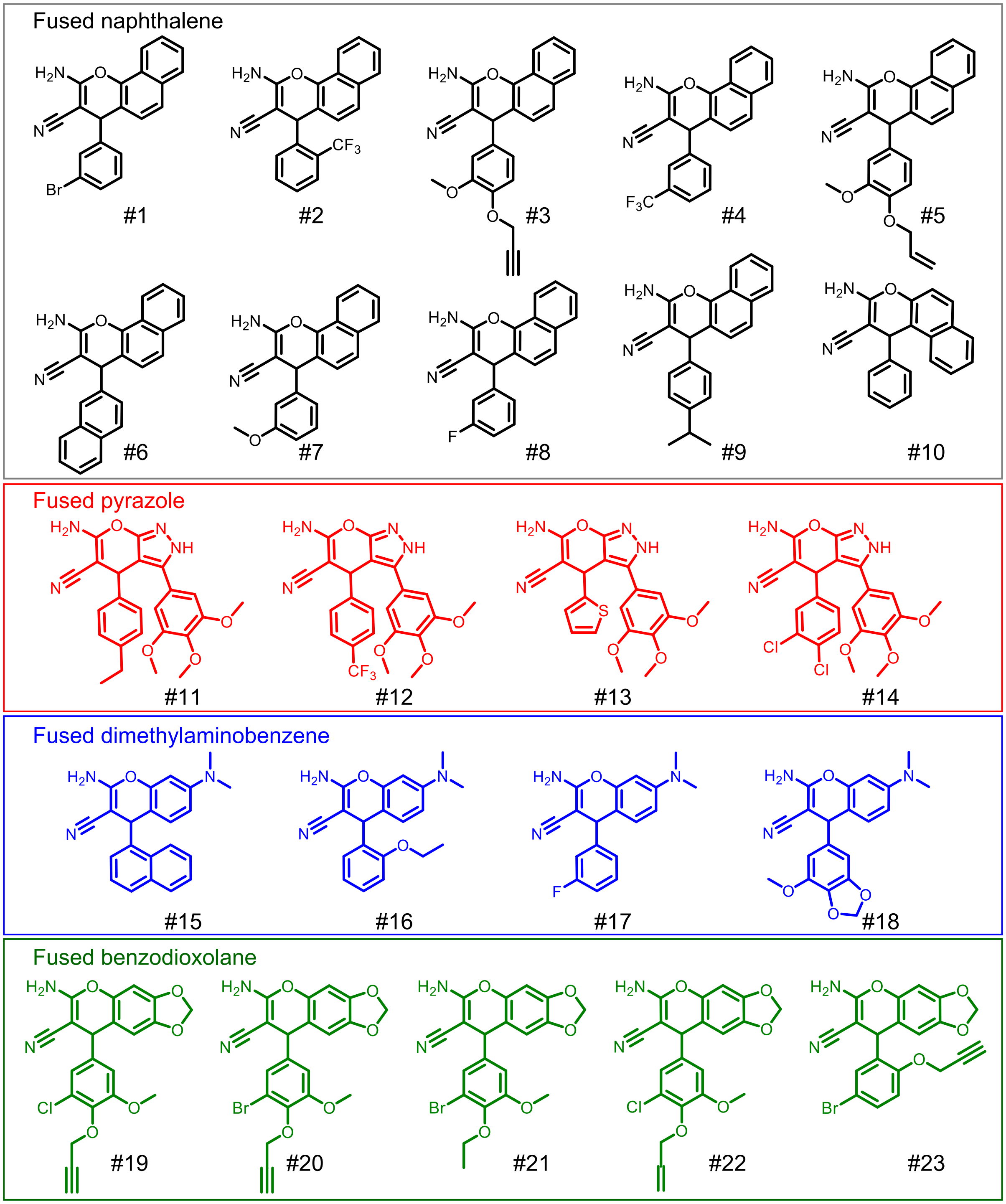

Supplementary Figure 2

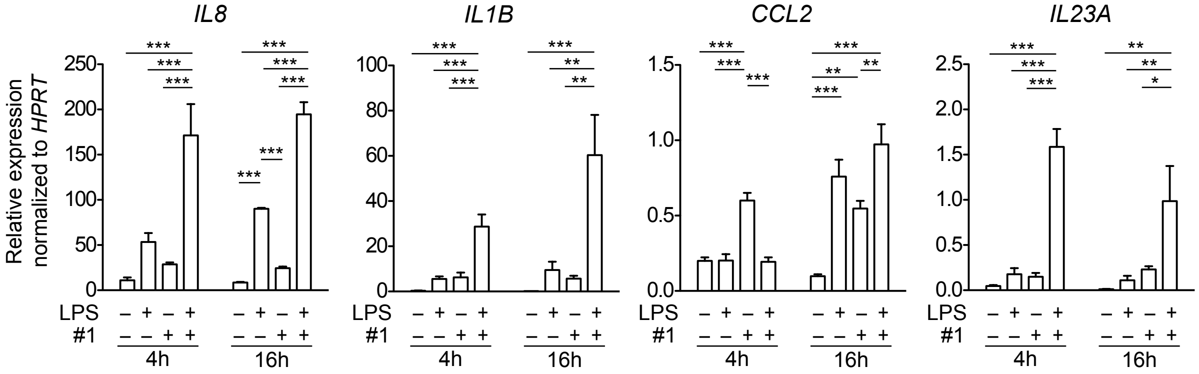

Supplementary Figure 3

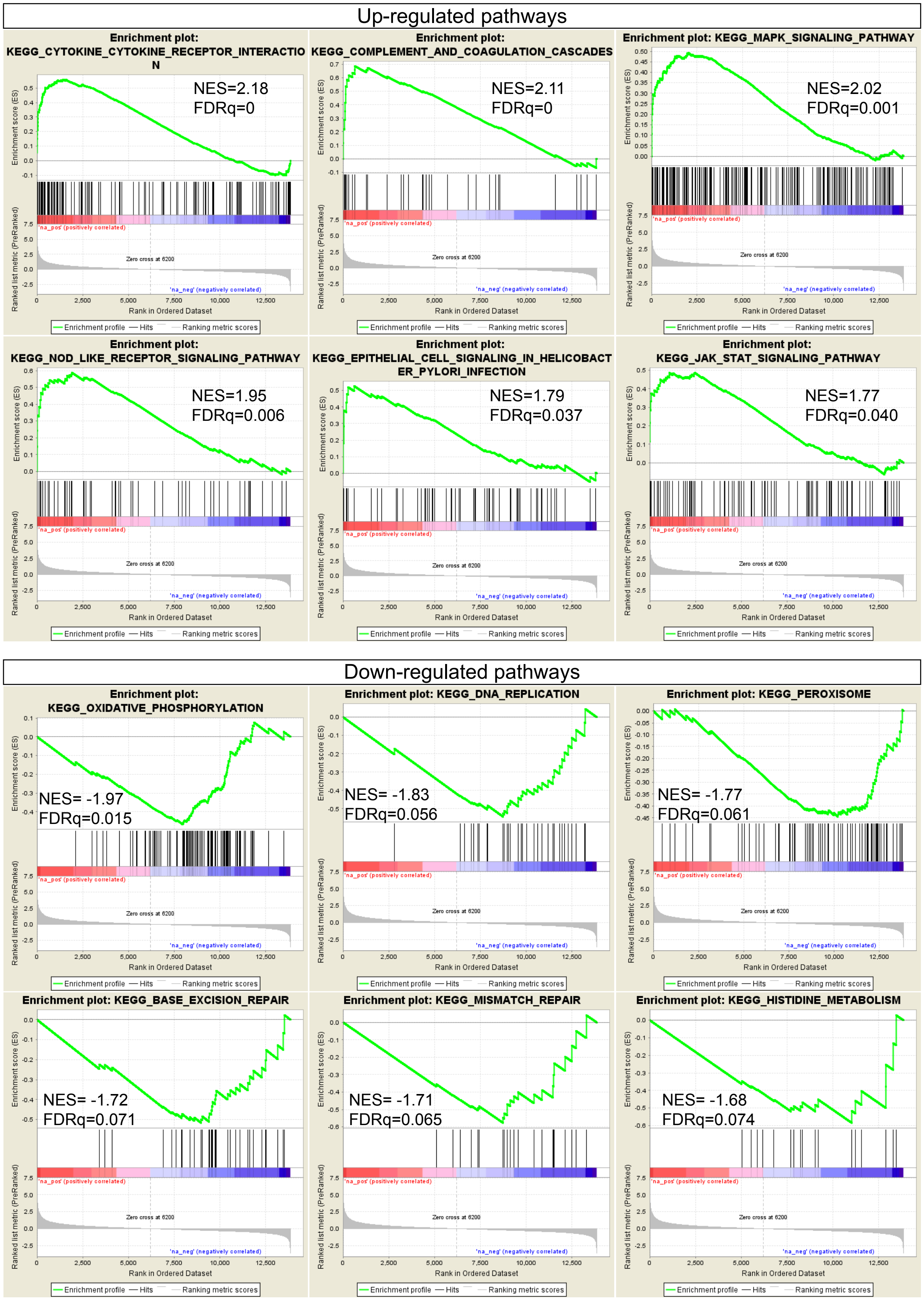

Supplementary Figure 4

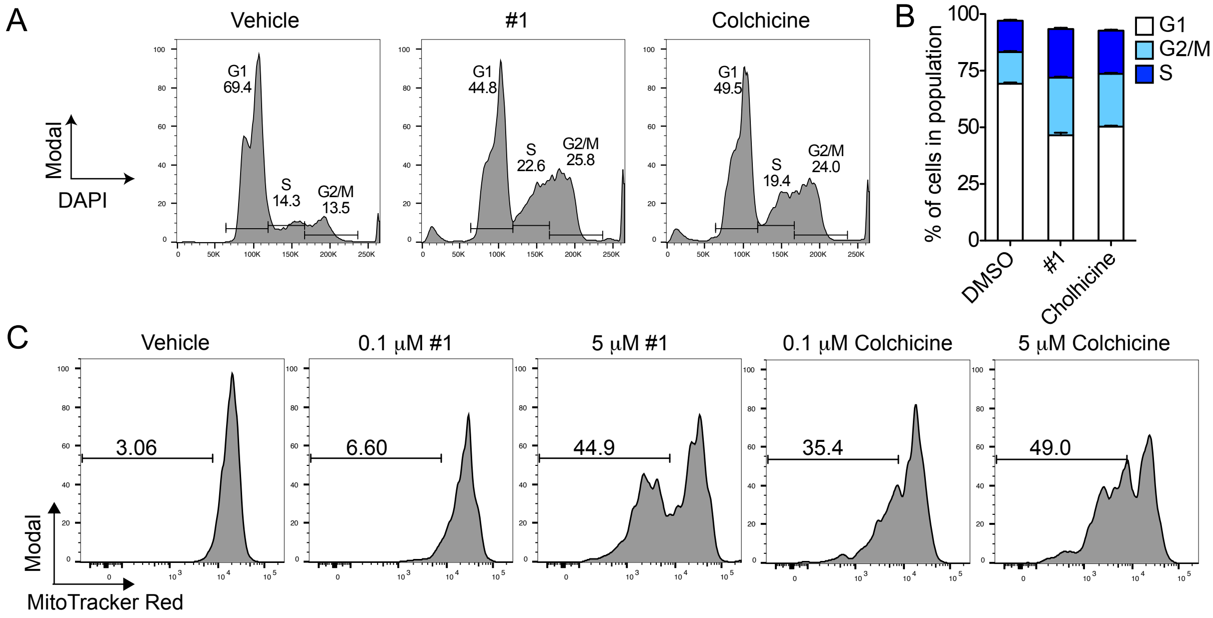

Supplementary Figure 5

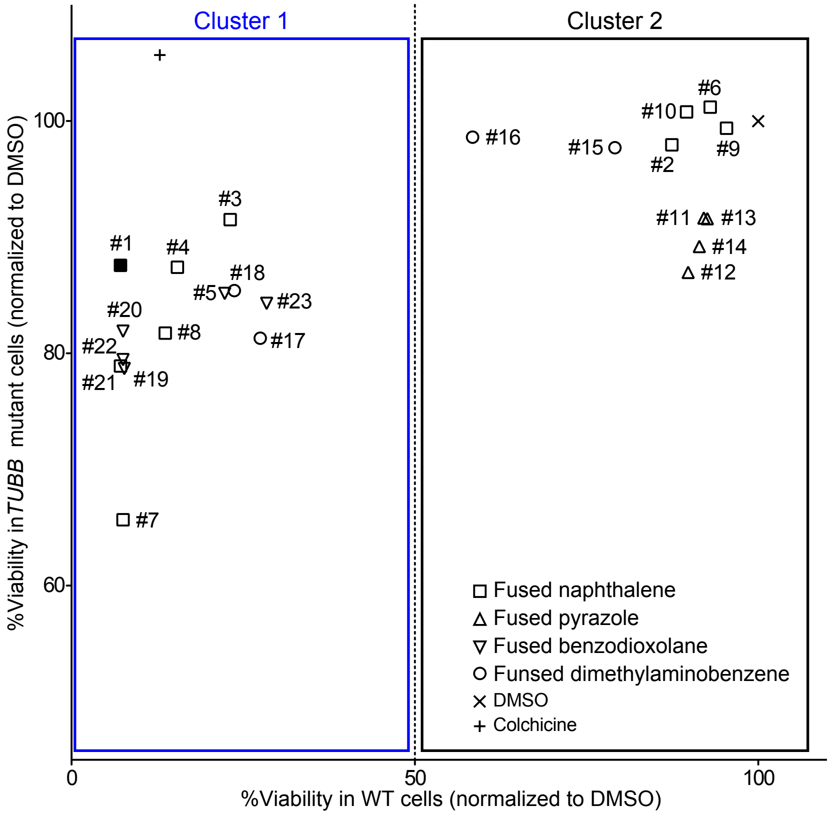

Supplementary Figure 6

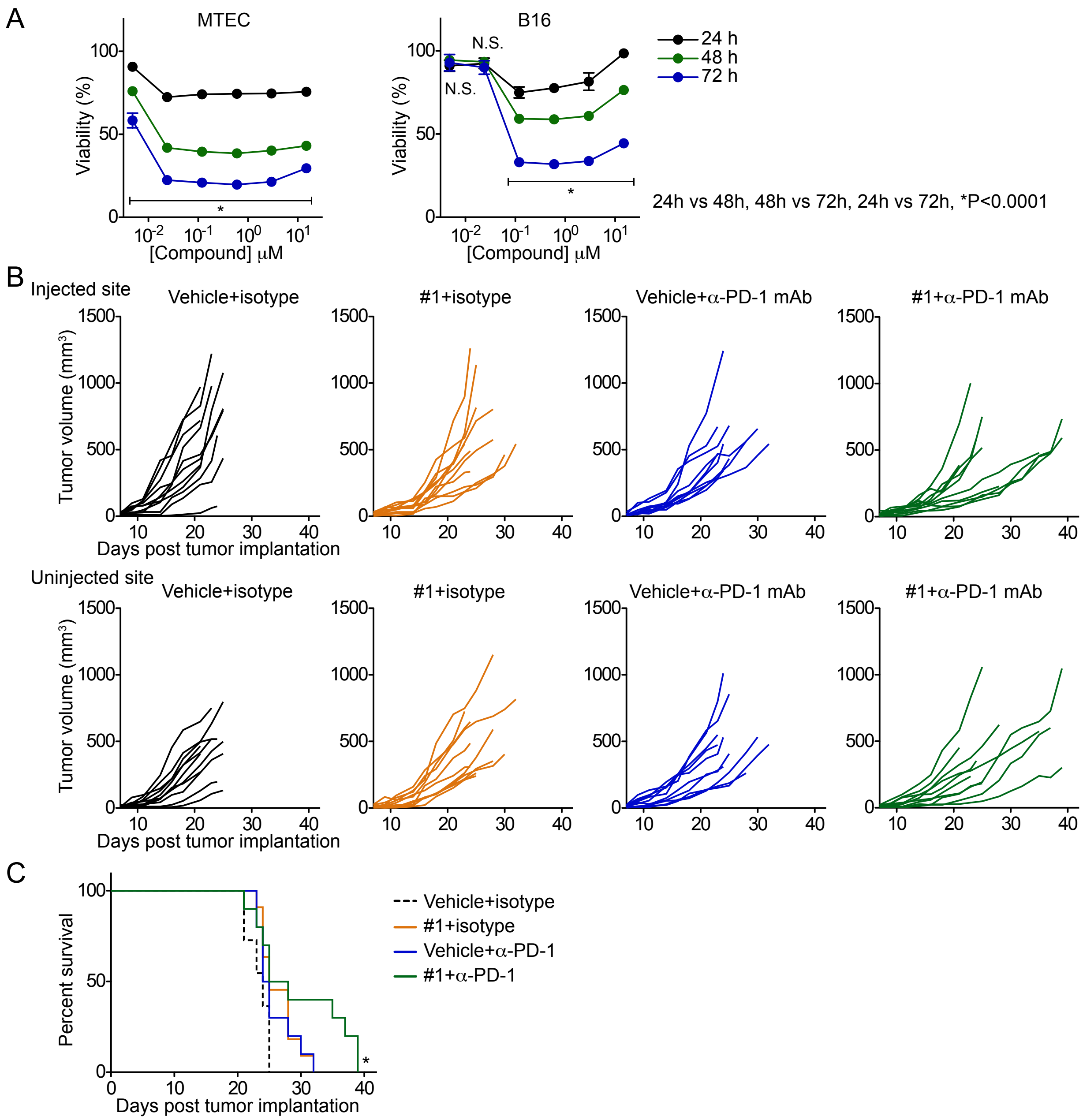

Supplementary Figure 7

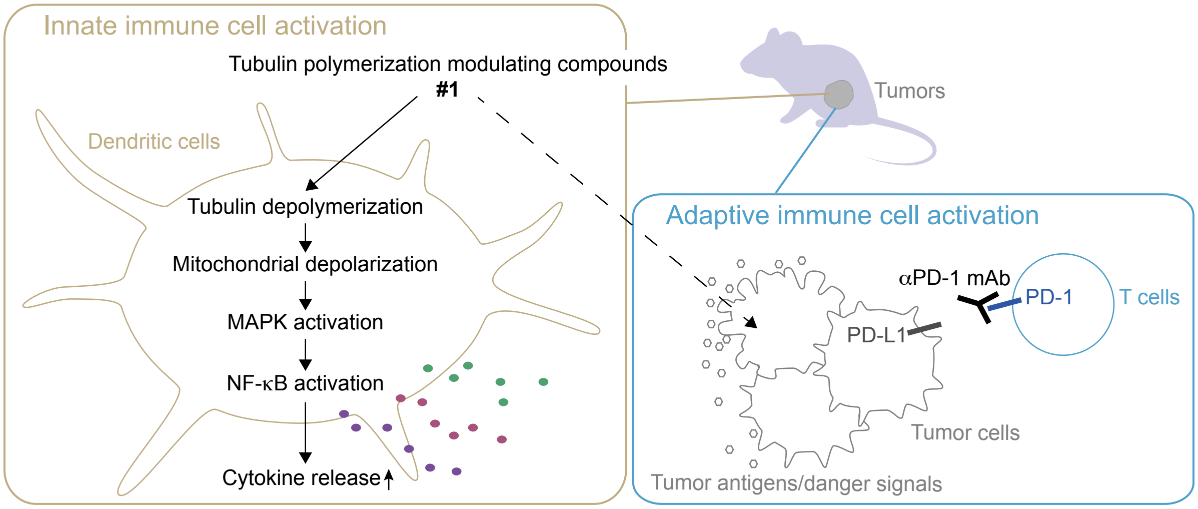

Supplementary Figures

Supplementary Figure 1. Structures of select purchased 4*H*-chromene-3-carbonitriles.

Supplementary Figure 2. Quantitative RT-PCR of gene expression of *IL8*, *IL1B*, *CCL2* and *IL23A* in THP-1 cells. THP-1 cells were treated with 5 μM **#1** in the presence and absence of LPS for 4 and 16 h. Expressions of *IL8*, *IL1B*, *CCL2* and *IL23A* were measured by quantitative RT-PCR and the levels were normalized to HPRT. $^{*}P<0.05$, $^{**}P<0.01$, $^{***}P<0.0001$ by one-way ANOVA with Tukey's *post hoc* test.

Supplementary Figure 3. RNA-seq data was analyzed by enrichment method using KEGG pathway database. THP-1 cells were incubated with compound **#1** for 5 h and RNA was isolated. RNA-seq was performed by La Jolla Institute Next Generation Sequencing Facility. The most significantly up-regulated (upper panels) and down-regulated pathways (lower panels) are shown (FDR $q<0.05$).

Supplementary Figure 4. Compound **#1** induces cell cycle arrest at the G2/M phase and mitochondrial depolarization in THP-1 cells. (A) Representative histograms of cell cycle analysis. 5×10^{4} THP-1 cells cultured with 5 μM **#1**, or colchicine overnight. After fixation with 70% ethanol, cells were washed with PBS and stained with 10 μg/ml of DAPI for 15 min at room temperature and flow cytometry analysis was performed. (B) Distribution (%) of cells in G1, G2/M and S phases. Data presented are representative of two independent experiments showing similar results. (C) THP-1 cells were treated with 0.1 or 5 μM **#1**, or colchicine for 72 h and stained with MitoTracker Red, which stains mitochondria in a membrane potential dependent manner. Cells were analyzed by flow cytometry and representative histograms are shown.

Supplementary Figure 5. Detailed compound distribution of Figure 4B. The individual compound IDs are labeled next to their respective symbols from the plot in Figure 4B.

Supplemental Figure 6. Anti-tumor effects of compound **#1**. (A) Cytotoxicity of **#1** increased in a time-dependent manner. 10^5 cells/mL HPV negative MTECs or B16-OVA cells were cultured for 24 h (black), 48 h (green) and 72 h (blue) with serially diluted compound **#1** from 15 μM. At the indicated times cell viability was tested by MTT assay as described in the methods. Comparisons among 24 h, 48 h and 72 h at each concentration were examined by one-way ANOVA with Tukey's *post hoc* test. (*P<0.0001, N.S.: not significant). (B) Tumor growth curves from individual mice in Figure 6F are shown. (C) Kaplan-Meier survival curves of each treatment group are shown. Mice that received combination treatment survived longer compared to vehicle treated mice [*P<0.05 by Log-rank (Mantel-Cox) test].

Supplemental Figure 7. Model of functional activity for compound **#1**. Compound **#1** inhibits intracellular beta-tubulin polymerization, which is associated with mitochondrial depolarization and cell cycle arrest. Mitochondrial distress initiates a cascade of MAPK and NF-κB activation which results in cytokine/chemokine release. Intratumor injection of compound **#1** activates APCs in the tumor microenvironment and concurrently induces the release of tumor-antigens, which provides the stimulus of tumor-specific cellular responses.

Supplementary Table 1. Substructure based classification of 4*H*-chromene-3-carbonitriles into major groups

Group	Fused scaffold name	Total # of compounds	NF-κB HTS [a]	ISRE HTS [a]
**1** [b]	**Fused naphthalene**	**41**	**1**	**8**
**2** [b]	**Fused pyrazole**	**706**	**8**	**1**
**3** [b]	**Fused benzodioxolane**	**12**	**1**	**3**
**4** [b]	**Fused dimethylaminobenzene**	**20**	**0**	**6**
5	Other substituted Fused Benzene	107	1	1
6	Fused 4-phenyl-5-carboxy	524	1	0
7	Fused 4-heteroaryl-5-carboxy	174	0	0
8	Fused pyrimidine	48	0	0
9	Fused imidazole	13	0	1
10	Fused piperidine	22	1	0
11	Fused quinoline	23	0	0
12	Fused thiophene	27	0	0
13	Fused pyridine	16	0	0
14	Others	45	0	0
Total		*1778*	*13*	*20*

[a] Number of compounds with % activation > 90% in individual screens.
[b] Four groups having more than 2 compounds with "% activation" values greater than 90% full activation in either of the HTS screens were selected.

Supplementary Table 2. Bioactivities for 4*H*-chromene-3-carbonitriles

Compound ID [a]	THP-1 NF-κB Reporter cells SEAP (μg/mL)		mBMDC IL-12 (ng/mL)
	without LPS	with LPS	with LPS
	Mean ± SEM [b]	Mean ± SEM [c]	Mean ± SEM [c]
**#1**	0.06 ± 0.01*	3.25 ± 0.45**	9.42 ± 0.42**
**#2**	0.03 ± 0	2.33 ± 0.34	2.87 ± 0.2
**#3**	0.05 ± 0.01	3.06 ± 0.31**	7.17 ± 0.62**
**#4**	0.03 ± 0	2.92 ± 0.4**	9.65 ± 0.82**
**#5**	0.09 ± 0.02**	3.6 ± 0.3**	5.6 ± 0.14**
**#6**	0.02 ± 0	2.25 ± 0.34	1.77 ± 0.06***
**#7**	0.05 ± 0.01	3.16 ± 0.35**	6.76 ± 0.13**
**#8**	0.05 ± 0.01	3.18 ± 0.23**	8.18 ± 0.13**
**#9**	0.02 ± 0	1.45 ± 0.23	1.34 ± 0.19***
**#10**	0.03 ± 0	1.82 ± 0.21	1.81 ± 0.21***
**#11**	0.02 ± 0	1.11 ± 0.11	2.05 ± 0.05
**#12**	0.02 ± 0	1.21 ± 0.11	0.99 ± 0.02***
**#13**	0.02 ± 0	1.85 ± 0.43	0.3 ± 0.02***
**#14**	0.02 ± 0	1.49 ± 0.42	0.68 ± 0.22***
**#15**	0.03 ± 0.01	2.65 ± 0.43*	2.41 ± 0.11
**#16**	0.03 ± 0.01	2.57 ± 0.42	2.19 ± 0.09***
**#17**	0.04 ± 0.01	2.88 ± 0.35**	2.84 ± 0.07
**#18**	0.06 ± 0.01	3.77 ± 0.35**	5.39 ± 0.1**
**#19**	0.09 ± 0.03*	3.52 ± 0.32**	6.68 ± 0.37**
**#20**	0.09 ± 0.03*	3.63 ± 0.41**	8.19 ± 0.46**
**#21**	0.08 ± 0.02	3.24 ± 0.24**	8.17 ± 0.08**
**#22**	0.04 ± 0.01	2.47 ± 0.22	2.69 ± 0.12***
**#23**	0.06 ± 0.01	3.61 ± 0.24**	8.52 ± 0.34
DMSO	0.02 ± 0	--	--
LPS	--	1.61 ± 0.19	2.85 ± 0.11

[a] Compounds were evaluated at 5 μM concentration
[b), c)] Statistical significance calculated using one-way ANOVA and Dunnett's multiple comparison test compared to DMSO [b] or LPS [c].
Significant increase is denoted as **, P < 0.01 or *, P < 0.05 and significant decrease is denoted as ***, P < 0.01.

Supplementary Table 3. EC_{50} values of selected compounds for enhanced IL-12 and IL-6 production with LPS co-stimulation

Compound ID	Subscaffold	IL-12 EC_{50} (μM)[a)]	IL-6 EC_{50} (μM)[a)]
**#1**	Fused napthalene	0.23	0.15
**#3**	Fused napthalene	1.78	1.98
**#4**	Fused napthalene	0.88	0.69
**#5**	Fused napthalene	1.91	2.75
**#7**	Fused napthalene	0.34	0.36
**#8**	Fused napthalene	0.89	0.79
**#18**	Fused dimethylaminobenzene	2.83	0.61
**#19**	Fused benzodioxolane	0.52	0.51
**#20**	Fused benzodioxolane	0.73	0.69
**#21**	Fused benzodioxolane	0.32	0.19
**#22**	Fused benzodioxolane	0.47	0.25

[a)] BMDCs were incubated with decreasing (1:2 dilutions) concentrations of compound (from 10μM) in the presence of 0.5 ng/mL LPS for 18 h. IL-12 and IL-6 secretion levels were evaluated by ELISA.

Supplementary Table 4. KEGG pathway analysis from RNA sequencing data

Up-regulated genes by #1 treatment compared to vehicle

NAME	# genes	ES	NES	NOM. p.val	FDR.q.val	#genes.pos	Genes
CYTOKINE_CYTOKINE_RECEPTOR_INTERACTION	135	0.56	2.18	0.00	0.00	46	LIF,IL8,OSM,IL1B,CXCL2,CCL20,TNFRSF12A,CXCL3,IL1A,IL23A,CCL4,INHBA,TNFRSF9,CXCL1,IL7R,VEGFA,TNFRSF10B,CCL3,TNFSF15,CXCL10,IL1RAP,IL10RA,TNFRSF18,TNF,FLT1,PRLR,PDGFA,IL1R1,CXCL16,IL4R,INHBE,CLCF1,TNFSF9,IL18,IFNGR1,IL2RG,TSLP,CXCR4,CCR7,CSF2RB,TNFRSF10D,ACVR2A,ACVR1,KITLG,TNFRSF1B,IL15
COMPLEMENT_AND_COAGULATION_CASCADES	35	0.69	2.11	0.00	0.00	12	BDKRB2,PLAU,SERPINE1,PLAUR,CD55,TFPI,C5AR1,C3AR1,THBD,SERPIND1,F3,MASP2
MAPK_SIGNALING_PATHWAY	203	0.49	2.02	0.00	0.00	57	DUSP,NR4A1,IL1B,DUSP8,DUSP2,DUSP4,JUN,IL1A,DUSP16,JUND,DUSP5,NFKB2,RELB,MAP3K14,DUSP10,PLA2G10,TNF,MAPK8IP1,MAP3K8,PDGFA,GADD45B,NFKB1,IL1R1,TRAF6,MAP2K3,CRK,PTPRR,KRAS,FGF5,GNG12,MAP3K2,FOS,BRAF,CACNA2D2,RPS6KA5,DUSP14,CACNB4,RASA2,GADD45A,MAPK7,DDIT3,PPM1A,RASGRF2,CACNA1F,MAPK8,CACNG8,FLNB,RASGRP1,PPM1B,PLA2G6,MAPKAPK2,SOS2,RAP1B,ATF4,CACNB2,ELK4,MAP3K12
NOD_LIKE_RECEPTOR_SIGNALING_PATHWAY	55	0.59	1.95	0.00	0.01	19	IL8,IL1B,CXCL2,BIRC3,NFKBIA,CXCL1,TNFAIP3,TNF,NFKB1,TRAF6,IL18,RIPK2,NLRP3,MAPK8,BIRC2,XIAP,NOD2,TRIP6,NFKBIB
EPITHELIAL_CELL_SIGNALING_IN_HELICOBACTER_PYLORI_INFECTION	60	0.53	1.79	0.00	0.04	7	IL8,HBEGF,JUN,NFKBIA,CXCL1,MAP3K14,NFKB1
JAK_STAT_SIGNALING_PATHWAY	88	0.49	1.77	0.00	0.04	15	LIF,OSM,SOCS3,IL23A,STAT4,IL7R,IL10RA,SPRED2,PRLR,PIK3R5,IL4R,CLCF1,IFNGR1,IL2RG,TSLP

ES is enrichment score, NES is normalized enrichment score, cut-off >=2. Order of gene names: high to lower expression, the most upregulated gene is the top.

Down-regulated genes by #1 treatment compared to vehicle

NAME	# genes	ES	NES	NOM. p.val	FDR.q.val	#genes.pos	Genes
OXIDATIVE_PHOSPHORYLATION	103	-0.47	-1.97	0.00	0.02	75	ATP6V1C1,ATP6V1B1,NDUFS5,NDUFS4,NDUFA6,ATP6V1A,NDUFB1,ATP6V0A2,ATP5C1,COX6C,COX5A,NDUFV3,NDUFB3,NDUFA1,NDUFB4,NDUFB2,COX6A1,ATP6V1G1,ATP6AP1,COX7C,SDHB,ATP5L,ATP5J,ATP5I,UQCRQ,COX4I1,UQCRFS1,NDUFB9,ATP5D,ATP6V1D,NDUFB8,ATP5J2,COX6B1,SDHC,ATP6V1E1,TCIRG1,COX15,COX5B,ATP5B,UQCR11,ATP5F1,ATP5A1,COX8A,NDUFS6,ATP5O,NDUFAB1,ATP6V0B,NDUFV1,NDUFV2,NDUFA3,NDUFA4,NDUFA9,COX11,NDUFB10,NDUFS2,NDUFS1,NDUFA2,ATP5G2,ATP6V0D1,ATP6V0E1,ATP5G1,NDUFS3,ATP5H,ATP5G3,UQCR10,NDUFS8,SDHA,NDUFA8,NDUFA11,NDUFB7,CYC1,NDUFS7,ATP5E,NDUFA4L2,ATP6V0E2
DNA_REPLICATION	35	-0.54	-1.83	0.00	0.06	25	RFC1,RPA2,SSBP1,POLD4,POLE3,RFC5,LIG1,RNASEH2C,POLE2,POLE,MCM7,PCNA,PRIM1,POLD1,MCM6,RFC2,POLD2,FEN1,MCM4,MCM5,POLA2,RNASEH2A,RPA1,MCM3,MCM2
PEROXISOME	70	-0.45	-1.77	0.00	0.06	34	SLC25A17,ABCD3,SCP2,CAT,CRAT,PRDX1,PXMP2,ACOT8,SLC27A2,ECH1,DDO,GSTK1,IDH2,MPV17L,PEX26,MLYCD,NUDT12,PEX3,PEX11B,PEX11G,DHRS4,HMGCL,ABCD1,PEX10,PEX12,PIPOX,PMVK,CROT,PEX11A,EHHADH,PXMP4,IDH1,PAOX,AMACR
BASE_EXCISION_REPAIR	33	-0.52	-1.72	0.00	0.07	23	POLD4,POLE3,XRCC1,POLB,HMGB1,LIG1,MPG,POLE2,NEIL3,POLE,PCNA,POLL,POLD1,LIG3,POLD2,OGG1,FEN1,APEX2,SMUG1,APEX1,UNG,NTHL1,PARP1
MISMATCH_REPAIR	22	-0.58	-1.71	0.00	0.07	16	RFC1,RPA2,SSBP1,POLD4,RFC5,EXO1,LIG1,PCNA,POLD1,RFC2,MLH3,POLD2,MSH2,MSH6,RPA1,PMS2
HISTIDINE_METABOLISM	18	-0.58	-1.68	0.00	0.07	7	ALDH3A2,ALDH9A1,LCMT2,ABP1,ALDH3B1,ACY3,ALDH1A3

ES is enrichment score, NES is normalized enrichment score, cut-off ≧ 2. Order of gene names: high to low expression, most down-regulated gene is the last
